# Validation of German Aortic Valve Score in a Multi-Surgeon Single Center

**DOI:** 10.21470/1678-9741-2016-0029

**Published:** 2017

**Authors:** Mehmet Kalender, Ahmet Nihat Baysal, Okay Guven Karaca, Kamil Boyacioglu, Nihan Kayalar

**Affiliations:** 1 Konya Education Research Hospital, Cardiovascular Surgery Department, Konya, Turkey.; 2 Duzce University Medical School Hospital, Cardiovascular Surgery Department, Duzce, Turkey.; 3 Bagcilar Education and Research Hospital, Cardiovascular Surgery Department, Istanbul, Turkey.

**Keywords:** Aortic Valve, Risk Assessment, Adult, Risk Grade

## Abstract

**Objective:**

Risk assessment for operative mortality is mandatory for all cardiac operations. For some operation types such as aortic valve repair, EuroSCORE II overestimates the mortality rate and a new scoring system (German AV score) has been developed for a more accurate assessment of operative risk. In this study, we aimed to validate German Aortic Valve Score in our clinic in patients undergoing isolated aortic valve replacement.

**Methods:**

A total of 35 patients who underwent isolated open aortic valve replacement between 2010 and 2013 were included. Patients with concomitant procedures and transcatheter aortic valve implantation were excluded. Patients' data were collected and analyzed retrospectively. Patients' risk scores EuroSCORE II were calculated online according to criteria described by EuroSCORE taskforce, Aortic Valve Scores were also calculated.

**Results:**

The mean age of patients was 61.14±13.25 years (range 29-80 years). The number of female patients was 14 (40%) and body mass index of 25 (71.43%) patients was in range of 22-35. Mean German Aortic Valve Score was 1.05±0.96 (min: 0 max: 4.98) and mean EuroSCORE was 2.30±2.60 (min: 0.62, max: 2.30). The Aortic Valve Score scale showed better discriminative capacity (AUC 0.647, 95% CI 0.439-0.854). The goodness of fit was x^2^HL=16.63; *P*=0.436). EuroSCORE II scale had shown less discriminative capacity (AUC 0.397, 95% CI 0.200-0.597). The goodness of fit was good for both scales. The goodness of fit was x^2^HL=30.10; *P*=0.610.

**Conclusion:**

In conclusion, German AV score applies to our population with high predictive accuracy and goodness of fit.

**Table:** 

Abbreviations, acronyms & symbols	
BMI	= Body mass index
EuroSCORE	= European System for Cardiac Operation Risk
	Evaluation
ROC	= Receiver operating characteristic
TAVI	= Transcatheter aortic valve implantation

## INTRODUCTION

The assessment of operative mortality risk is mandatory for all cardiac operations. Patients need to be informed preoperatively about the risk factors. Some risk scoring systems are used to compare and standardize the results of the operations. The European System for Cardiac Operation Risk Evaluation (EuroSCORE) is a risk model published in 1999^[1]^. For more than a decade, this risk model had been used widely and validated in innumerable papers demonstrating wonderful goodness of fit^[2,3]^. Current requirements necessitated an update to scoring systems which ended up developing EuroSCORE II which was published on May 2010^[2]^. EuroSCORE II also demonstrated a discriminative capacity similar to EuroSCORE (AUC _EuroSCORE II_=0.81 *vs.* AUC _EuroSCORE_=0.78), and good calibration (x^2^_HL_=15.48; *P*=0.0505)^[4]^. On the other hand, for specific operation types such as aortic valve repair, EuroSCORE II overestimates the mortality rate^[5-7]^ which resulted in development of a new scoring system. Some of these new scoring systems emerged nation based such as Ambler, Guaragna and German Aortic Valve score (formerly named AKL-score)^[8-10]^. German Aortic Valve Score was described by Kötting et al.^[10]^ in 2013 with a study in which 1147 isolated aortic valve surgery and transcatheter aortic valve implantation (TAVI) patients were enrolled. German aortic valve score has 15 risk factors (Table 1). Two of them (body mass index - BMI - and no sinus rhythm) are different from EuroSCORE II. EuroSCORE II differs in five parameters comparing to German Aortic Valve score (hand poor mobility, diabetes on insulin, Canadian Cardiovascular Society class 4 angina, weight of the intervention and thoracic aorta surgery) - Table 2.

**Table 1:** Patients' characteristics. German Aortic Valve Score.

		n	%	Mortality
Age group (years)	<66	20	57.14	5
	66-70	5	14.29	0
	71-75	7	20	1
	76-80	3	8.571	0
Sex	Male	21	60	5
	Female	14	40	1
BMI	22-35	25	71.43	4
	<22	8	22.86	2
	>35	2	5.71	0
Heart failure: NYHA IV	NYHA<IV	34	97.14	6
	NYHA=IV	1	2.85	0
Myocardial infarction < 3 weeks		0	0	0
Critical preoperative status		0	0	0
Pulmonary hypertension		13	37.14	3
No sinus rhythm		4	11.43	1
LVEF (%)	<30	1	2.857	0
	30-50	10	28.57	1
	>50	24	68.57	5
Endocarditis		1	2.85	0
Reoperation		1	2.85	0
Peripheral arterial disease		0	0	0
Chronic obstructive pulmonary disease		5	14.29	1
Chronic renal insufficiency		2	5.714	1
Emergency		2	5.714	0
Observed mortality		6	17.14	6

BMI = body mass index; LVEF = left ventricular ejection fraction; NYHA = New York Heart Association

**Table 2:** Patients' characteristics. EuroSCORE II.

	n	%	Mortality
Patient related factors			
Age (years)		61.14±13.25	6
Female	14	40	1
Peripheral arteriopathy	0	0	0
Chronic obstructive pulmonary disease	5	14.3	1
Diabetes on insulin	3	8.6	0
Poor mobility	0	0	0
Renal impairment			
Dialysis	2	5.71	1
CC<50	5	14.28	0
85<CC>50	20	57.14	2
CC>85	8	22.85	2
Cardiac related factors			
Active endocarditis	1	2.9	0
Recent AMI	0	0	0
NYHA class			
II	31	88.4	4
III	3	8.7	2
IV	1	2.9	0
CCS4	0	0	
LVEF (%)			
>50	24	68.57	5
31-50	10	28.57	1
21-30	1	2.85	0
<20	0	0	0
Pulmonary artery pressure			
31-55 mmHg	3	8.7	0
>55 mmHg	1	2.9	1
Procedure			
Critical Condition	0	0	0
Re-operation	1	2.9	1
Thoracic aorta	0	0	0
Emergency			
Urgent	0	0	0
Emergent	2	5.7	0
Salvage	0	0	0
Weight of procedure			
Single non-CABG	35	100	6

AMI = acute myocardial infarction; CABG = coronary artery bypass grafting; CCS = Canadian Cardiovascular Society; LVEF = left ventricular ejection fraction; NYHA = New York Heart Association

In this study, we aimed to validate German Aortic Valve Score by comparing it with original the EuroSCORE II risk scoring system in patients with isolated open aortic valve replacement.

## METHODS

Patients who underwent isolated open aortic valve replacement between May 2010 and June 2013 were included in the study. Those with concomitant procedures, isolated bioprosthesis replaced patients and TAVI were excluded. Patients' data were collected and analyzed retrospectively. Primary end point was observed in hospital mortality. Patients' risk scores EuroSCORE II were calculated online according to criteria described by EuroSCORE taskforce^[11]^. Aortic Valve Scores were calculated according to criteria described by Kötting et al.^[10]^.

Sensitivity and specificity was assessed by the use of receiver operating characteristic (ROC) curve and the calibration of German Aortic Valve Score was assessed by Hosmer-Lemeshow (HL) test^[12]^. Calibration was considered to be poor if the test was significant. The discrimination measures the capacity of a model (in this case German Aortic Valve Score and EuroSCORE II) to differentiate the individuals of a sample that suffer an event (in this case, death) and those that do not. The discriminative capacity of the analyzed event was estimated by mean of ROC curve^[13]^. For the analysis, the statistical package SPSS^®^ 15.0 (SPSS, Inc., Chicago, IL, USA) for Windows^®^ was used. A *P*-value <0.05 was considered significant.

## RESULTS

We evaluated 35 isolated aortic valve replacement operations in adult patients for this study. The mean age of patients was 61.14±13.25 years (range 29-80 years). The number of female patients was 14 (40%). Patients' characteristics are shown in Tables 1 and 2.

**Fig. 1:**
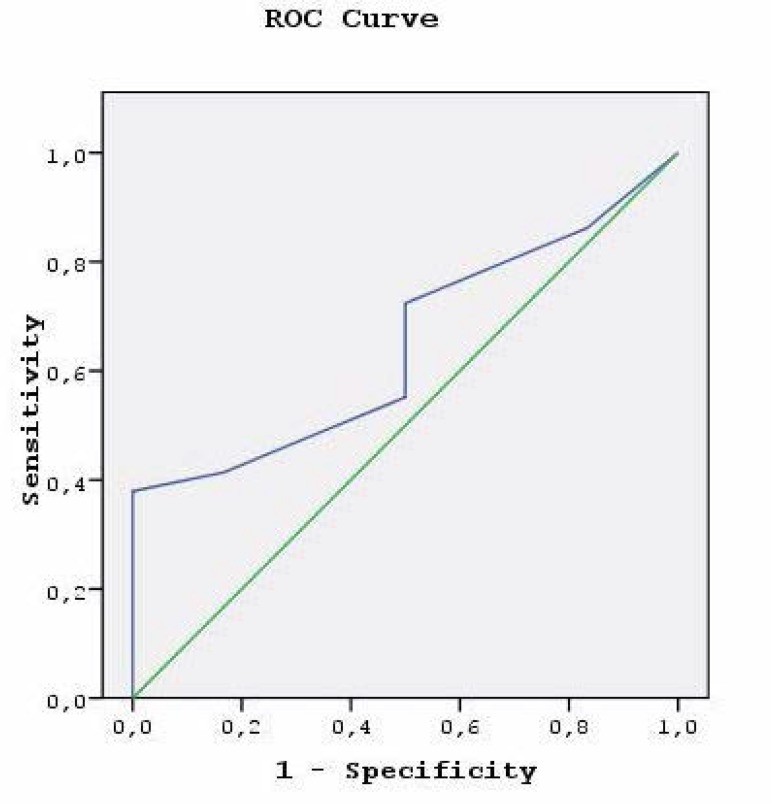
The receiver operating characteristic (ROC) curve of German aortic valve score.

**Fig. 2:**
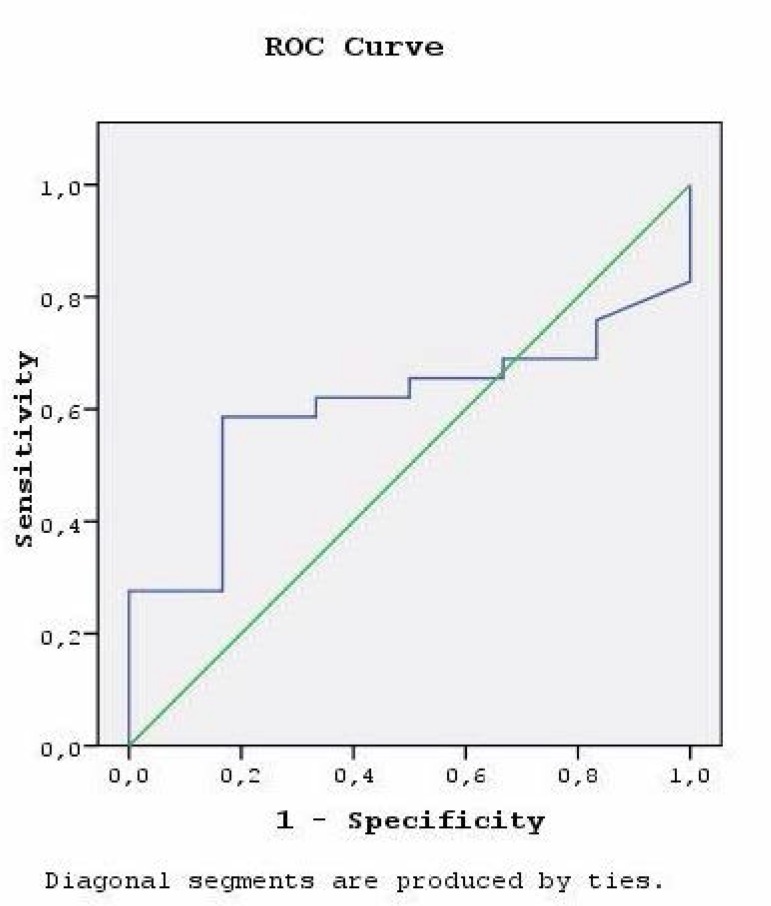
The receiver operating characteristic (ROC) curve of EuroSCORE II.

Mean German Aortic Valve Score was 1.05±0.96 (min: 0, max: 4.98) and mean EuroSCORE was 2.30±2.60 (min: 0.62, max: 2.30). The Aortic Valve Score scale showed better discriminative capacity (AUC 0.647, 95% CI 0.439-0.854) (Figure 1). The goodness of fit was x^2^_HL_=16.63; *P*=0.436) (Table 3). EuroSCORE II scale had shown less discriminative capacity (AUC 0.397, 95% CI 0.200-0.597) (Figure 2). The goodness of fit was good for both scales. The goodness of fit was x2HL=30.10; P=0.610 (Table 4).

**Table 3:** Contingency table for Hosmer–Lemeshow test (German Aortic Valve Score).

	Observed mortality = 0		Observed mortality = 1		Total
	Observed	Expected	Observed	Expected	Observed
1	4	3.967	0	33	4
2	4	3.778	0	222	4
3	4	4.423	1	577	5
4	4	4.916	2	1.084	6
5	5	3.939	0	1.061	5
6	4	4.514	2	1.486	6
7	4	3.462	1	1.538	5

**Table 4:** Contingency table for Hosmer–Lemeshow test (EuroSCORE II).

	Observed mortality = 0		Observed mortality = 1		Total
	Observed	Expected	Observed	Expected	Observed
1	4	3.972	0	0.028	4
2	4	3.763	0	0.237	4
3	4	3.633	0	0.367	4
4	4	3.443	0	0.557	4
5	1	3.258	3	0.742	4
6	3	3.145	1	0.855	4
7	3	3.003	1	0.997	4
8	4	3.458	1	1.542	5
9	2	1.326	0	0.674	2

## DISCUSSION

Risk scoring systems are valuable for benchmarking of institution results, however, several risk scoring systems have been developed and used. EuroSCORE II is a new updated scoring system with better mortality score and goodness of fit. But some statistical questions have been raised recently^[14,15]^. Moreover, parallel to our opinion there are papers advocating that one scoring system for all patient groups, cardiac diseases and therapies can certainly be misleading^[10,16-18]^. EuroSCORE II was also based on a data set consisting mainly of coronary procedures. Therefore, we believe that there is a requirement for a new scoring system more adaptive for aortic valve procedures. There are also papers reporting the requirement of a new scoring system for aortic valve procedures^[8,10,19-21]^. Kotting et al.^[10]^ described a new sco ring system for aortic valve procedures based on German Registry.

Former predictive models were developed for specific locations , but global need made EuroSCORE and STS popular and they were used widely. As Casalino et al.^[22]^ reported in their study that German Aortic Valve Score best fits in German population, but in our opinion it may be applicable to our population as well. Our results showed a high quality of discrimination AUC 0.647 and Hosmer-Lemeshow method exhibited sufficient concordance in the predicted and observed mortality (x^2^_HL_=16.63; P=0.436).

Non-randomized and retrospectively design, single institution setting, multi-surgeon operations and small sample size were the major limitations of our study.

## CONCLUSION

In conclusion, German Aortic Valve score applies to our population with high predictive accuracy and goodness of fit.

**Table:** 

Authors' roles & responsibilities	
MK	Conception and study design; realization of operations; analysis and/or data interpretation; statistical analysis; manuscript redaction or critical review of its content; final manuscript approval
ANB	Conception and study design; realization of operations; analysis and/or data interpretation; statistical analysis; manuscript redaction or critical review of its content; final manuscript approval
OGK	Conception and study design; realization of operations; analysis and/or data interpretation; statistical analysis; manuscript redaction or critical review of its content; final manuscript approval
KB	Conception and study design; realization of operations; analysis and/or data interpretation; statistical analysis; manuscript redaction or critical review of its content; final manuscript approval
NK	Conception and study design; realization of operations; analysis and/or data interpretation; statistical analysis; manuscript redaction or critical review of its content; final manuscript approval
